# Endometrioid Carcinomas of the Ovaries and Endometrium Involving Endocervical Polyps: Comprehensive Clinicopathological Analyses

**DOI:** 10.3390/diagnostics12102339

**Published:** 2022-09-27

**Authors:** Jihee Sohn, Yurimi Lee, Hyun-Soo Kim

**Affiliations:** Department of Pathology and Translational Genomics, Samsung Medical Center, Sungkyunkwan University School of Medicine, Seoul 06351, Korea

**Keywords:** cervix, endocervical polyp, ovary, endometrium, endometrioid carcinoma, implantation metastasis

## Abstract

While synchronous ovarian and endometrial endometrioid carcinomas (ECs) have long been described in the literature, ovarian or endometrial EC involving concomitant endocervical polyp (ECP) has not yet been reported. This study aimed to investigate the histological types and prevalence of gynecological tumors co-existing with ECP and to comprehensively analyze the clinicopathological characteristics of ovarian and endometrial ECs involving ECPs. We searched for ECP cases associated with premalignant lesions or malignancies of the female genital tract occurring between March 2019 and February 2022. We then investigated the histological types and prevalence of gynecological tumors co-existing with ECP. In addition, we reviewed electronic medical records and pathology slides to collect the clinicopathological features of four patients with ovarian or endometrial EC involving ECP. We found 429 ECPs over the three-year study period. Of these, 68 (15.9%) were associated with premalignant or malignant lesions occurring in the uterine cervix, endometrium, and ovaries. Four of these cases, including two (0.5%) ovarian grade 3 ECs and two (0.5%) endometrial grade 1 ECs, involved ECPs. In the former cases (cases 1 and 2), ECs involving ECPs exhibited similar morphology and immunohistochemical staining results to those of advanced-stage ovarian EC. In the latter cases (cases 3 and 4), the histological and immunophenotypical features of EC involving ECP were identical to those of primary endometrial EC, despite the lack of tumor involvement in the myometrium, lower uterine segment, and cervical stroma as well as the absence of lymphovascular invasion and lymph node metastasis. In all cases, no evidence of benign endometriosis, endometrial hyperplasia without atypia, or atypical hyperplasia/endometrial intraepithelial neoplasm within ECP or the adjacent endocervical tissue was noted. Considering our results, the involvement of ECP by EC may have been caused by an implantation metastasis from the ovarian (cases 1 and 2) or endometrial (cases 3 and 4) EC. To the best of our knowledge, this is the first exploration of the synchronous occurrence of endometrial or ovarian EC and ECP involvement. Implantation metastasis via transtubal and trans-endometrial cavity migration may have been the pathogenic mechanism of ECP involvement.

## 1. Introduction

Endocervical polyps (ECPs) are benign proliferative lesions of the uterine cervix that typically occur in the endocervical canal [1]. Stromal overgrowth and reactive epithelial hyperplasia associated with repeated episodes of inflammation, an abnormal local response to increased estrogen levels, and the local congestion of cervical stromal blood vessels are involved in the development of ECP [1,2]. More than half of the patients with ECP are asymptomatic, with the ECPs being discovered incidentally during routine gynecological examinations. However, symptomatic ECPs manifest as vaginal discharge, leukorrhea, menorrhagia, metrorrhagia, postcoital bleeding, and postmenopausal bleeding [2].

Endometrioid carcinoma (EC) of the endometrium is the most common histological type of endometrial carcinoma. The microscopic appearance of this tumor resembles that of a normal proliferative-phase endometrium, with variable degrees of glandular complexity and nuclear pleomorphism [3]. The diagnosis of endometrial EC is usually based on clinical findings and imaging and confirmed with endometrial curettage. In addition, EC of the ovaries accounts for 15% of epithelial ovarian carcinomas [4] and is associated with endometriosis in most cases [5,6]. Ovarian EC, morphologically similar to endometrial EC, is characterized by a confluent back-to-back arrangement of variable-sized glands lined by pseudostratified columnar epithelium with elongated nuclei [7,8,9].

Primary EC of the uterine cervix has often been misclassified in the literature due to the lack of clear-cut diagnostic criteria [10]. Based on a new classification system, the International Endocervical Adenocarcinoma Criteria and Classification [11,12,13], cervical EC is a rare histological type of human papillomavirus (HPV)-independent endocervical adenocarcinoma (EAC), with an overall prevalence of 1.1% [13]. Determining whether EC has occurred in the uterine corpus or the cervix is difficult, but this distinction is important as the optimal management of EC and EAC differs significantly.

Synchronous endometrial and ovarian ECs have long been described in the literature. However, the synchronous occurrence of either endometrial or ovarian EC and ECP involvement has not yet been reported. This phenomenon poses diagnostic challenges, as determining whether they are independent primary tumors or metastases of each other, as well as what the original tumor may be, is difficult [14]. Clinical presentation, previous gynecological history, and ancillary tests, such as immunohistochemical staining, are helpful in correctly diagnosing morphologically challenging cases. An accurate pathological diagnosis is fundamental for proper clinical practices and optimal patient outcomes.

We recently encountered four cases of ovarian or endometrial EC involving ECP. The reported prevalence of malignancies involving ECPs is 0.1–0.5% [1], and a thorough literature review revealed no information about EC involving ECP. In this study, we investigated the histological types and prevalence of gynecological tumors co-existing with ECP. We then comprehensively analyzed the pathological characteristics of four patients with ovarian or endometrial EC involving ECP and discussed their clinical significance and the possible pathogenesis. Our observations may encourage pathologists to recognize and accurately diagnose this rare but distinct occurrence.

## 2. Materials and Methods

### 2.1. Case Selection

We searched in the departmental archives for cases matching the keywords “endocervical polyp” and “cervical polyp” occurring between March 2019 and February 2022. A total of 429 cases of ECP were included in this study, of which 68 (15.9%) were ECPs co-existing with premalignant lesions or malignancies arising in any site of the gynecological tract and 4 (0.9%) were microscopically identified areas of EC involving the surface and stroma of ECPs. Representative formalin-fixed, paraffin-embedded tissue blocks were used for immunohistochemical staining.

### 2.2. Clinical Data Collection

The following clinical information was obtained from electronic medical records and pathology reports: age of patients, presenting symptoms, previous gynecological history, preoperative imaging findings, clinical impressions, cervical punch biopsy results, history of endocervical curettage and endometrial curettage, data on neoadjuvant chemotherapy use, surgical procedures, final pathological diagnosis, International Federation of Gynecology and Obstetrics (FIGO) stages [15], data on postoperative treatment and recurrence, disease-free survival, treatments for recurrence, survival status, and overall survival.

### 2.3. Pathological Data Collection

Pathological information was obtained from the slide review. For ECs involving ECP, histological type and grade [16], the greatest dimension, invasion depth into the polyp stroma, endocervical glandular extension, polypectomy resection margin involvement, the presence of endometriosis, atypical hyperplasia/endometrial intraepithelial neoplasm (AH/EIN), and lymphovascular invasion (LVI) were collected. For endometrial ECs, histological type and grade [16]; the largest dimension; myometrial invasion depth; cervical stromal extension; involvement of the lower uterine segment, uterine serosa, and parametrium; lymph node metastasis; and adnexal extension were collected. For ovarian ECs, histological type and grade [16]; the largest dimension; ovarian surface extension; involvement of the salpinx, uterus, and peritoneum; lymph node metastasis; and the presence of ovarian endometriotic cyst were collected.

### 2.4. Immunohistochemical Staining

We performed immunohistochemical staining based on previously described methods [17,18,19,20,21,22]. Immunohistochemical staining was performed using the Bond Polymer Intense Detection System (Leica Biosystems, Buffalo Grove, IL, USA). Briefly, 4-μm-thick sections cut from formalin-fixed, paraffin-embedded tissue blocks were deparaffinized in xylene and rehydrated through a series of graded alcohols. After antigen retrieval, endogenous peroxidases were quenched with hydrogen peroxide. The sections were incubated with primary antibodies against estrogen receptor (ER, 1:150, clone 6F11, catalog number ER-6F11-L-CE, Novocastra, Leica Biosystems, Buffalo Grove, IL, USA), progesterone receptor (PR, 1:100, clone 16, catalog number PGR-312-L-CE, Novocastra, Leica Biosystems, Buffalo Grove, IL, USA), p16 (prediluted, clone E6H4, catalog number 805-4713, Ventana Medical Systems, Oro Valley, AZ, USA), p53 (1:200, clone DO-7, catalog number NCL-L-p53-DO7, Novocastra, Leica Biosystems, Buffalo Grove, IL, USA), and Wilms tumor 1 (WT1, 1:800, clone 6F-H2, catalog number 348M-98, Cell Marque, Rocklin, CA, USA). A biotin-free polymeric horseradish peroxidase-linker antibody conjugate system was used with a BOND-MAX automated immunostainer (Leica Biosystems, Buffalo Grove, IL, USA). After chromogenic visualization using 3,3′-diaminobenzidine, the sections were counterstained with hematoxylin, dehydrated in graded alcohols and xylene, and then embedded in a mounting solution. Appropriate controls were stained concurrently. Positive controls were luminal A-type invasive breast carcinoma for ER and PR; cervical high-grade squamous intraepithelial lesion (HSIL) for p16; and ovarian high-grade serous carcinoma for p53 and WT1. Negative controls were prepared by substituting non-immune serum for primary antibodies, which resulted in undetectable staining.

### 2.5. Immunohistochemical Interpretation

Each immunostained slide was scored by two pathologists [18,23,24,25,26,27,28,29]. Staining intensities of hormone receptors and WT1 were designated as either weak, moderate, or strong, and staining proportions were determined in increments of 5% across a 0–100% range and classified as negative, focal (<50%), or diffuse (≥50%). Expression patterns of p16 were considered diffuse and strong positivity when p16 was expressed in the nuclei and/or cytoplasm with continuous and strong staining. All other p16 expression patterns, including focal nuclear staining, wispy, blob-like, puddled, and scattered cytoplasmic staining, were interpreted as patchy positivity [1,22,23,25,28]. Similarly, p53 expression patterns were considered aberrant when any of the following features were observed: diffuse and strong nuclear immunoreactivity in ≥75% of tumor cells (i.e., over-expression); no nuclear immunoreactivity in any tumor cell (i.e., complete absence); or unequivocal cytoplasmic staining (i.e., cytoplasmic pattern). Immunostained slides exhibiting a variable proportion of tumor cell nuclei expressing p53 with mild-to-moderate intensity were considered wild type [30].

## 3. Results

### 3.1. The Prevalence and Histological Types of Gynecological Tumors Co-Existing with ECP

As shown in Table 1, of the 429 patients with ECP identified, 68 (15.9%) had premalignant or malignant lesions arising in the uterine cervix, endometrium, and ovaries. Most of the cervical tumors (17/20; 85.0%) involved ECP, with HSIL being the most common (11/17; 64.7%). Additionally, three of the five patients who underwent radical hysterectomy for squamous cell carcinomas (SCCs) had ECPs, and endocervical adenocarcinoma in situ (AIS) involving ECP was present in two patients. We also found one case of stratified mucin-producing intraepithelial lesion (SMILE) co-existing with HSIL, in which ECP was involved by both SMILE and HSIL. ECPs presented as separate lesions in one patient with HSIL and AIS and two patients with SCC.

In contrast with the cervical tumors, most of the endometrial and ovarian tumors associated with ECP did not directly involve the polyps. The most common endometrial tumor associated with ECP was EC (16/24; 66.7%), while 14 of the 16 endometrial EC cases simply co-existed with ECPs in the hysterectomy specimens, without direct involvement. Moreover, six ECP cases with endometrial AH/EIN had no direct involvement with the tumor, and 14 patients who underwent debulking surgery for ovarian high-grade serous carcinoma (HGSC) showed benign incidental ECPs in their hysterectomy specimens. Three clear cell carcinomas, two ECs, two mucinous carcinomas, and one low-grade serous carcinoma of the ovary also co-existed with ECP, without polyp or uterine cervix involvement. Therefore, two cases of endometrial EC and two cases of ovarian EC involving ECPs were ultimately detected. We investigated the clinicopathological features of four patients with ovarian or endometrial EC involving ECP.

### 3.2. Clinical Presentations of Four Patients with EC Involving ECP

**Figure 1:**
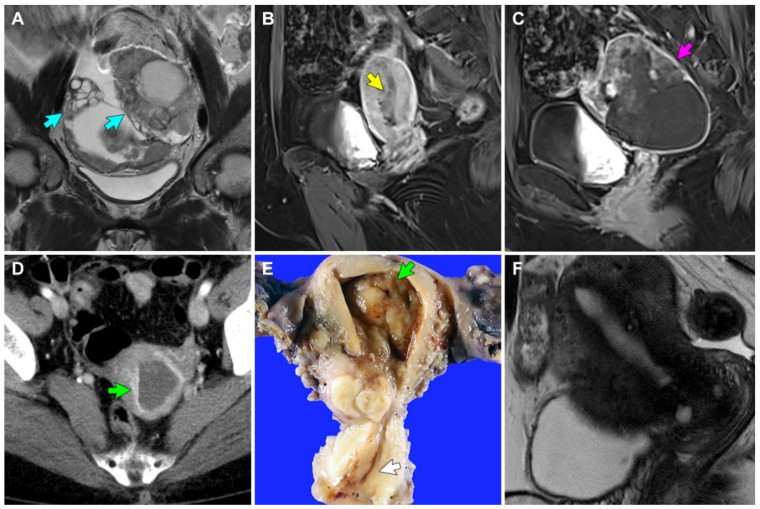
MRI, CT, and gross findings. (**A**) Case 1: T2-weighted coronal MR image reveals solid and cystic bilateral ovarian masses (blue arrows). (**B**,**C**) Case 2: T1-weighted Dixon sagittal MR images reveal (**B**) 3.4 cm endometrial mass (yellow arrow) and (**C**) 8.6 cm solid and cystic left ovarian mass (purple arrow). (**D**) Case 3: Contrast-enhanced CT image reveals a 1 cm endometrial mass (green arrow) and hematometra. (**E**) Case 3: An irregularly elevated mass is noted in the endometrium. The endometrial cavity is distended with blood. The endocervix (white arrow) appears unremarkable. (**F**) Case 4: T2-weighted sagittal MR image reveals no identifiable lesion in the endometrium.

*Case 1:* A 33-year-old woman with polycystic ovary syndrome was referred to our institution for the evaluation and management of adnexal masses, after visiting an outside hospital with lower abdominal discomfort. Abdominopelvic computed tomography (CT) and magnetic resonance imaging (MRI) revealed an 8.6 cm solid and cystic left ovarian mass and a 4.0 cm solid right ovarian mass (Figure 1A). An omental cake was noted, and some metastatic nodules were also identified in the cul-de-sac and right round ligament. Additionally, the retroperitoneal lymph nodes were mildly enlarged, and the endometrium was unremarkable. Following three cycles of neoadjuvant chemotherapy with paclitaxel and carboplatin, the patient underwent interval debulking surgery, including a total hysterectomy with bilateral salpingo-oophorectomy, pelvic and para-aortic lymph node dissection, omentectomy, peritoneal mass excision, and low anterior resection. The final pathological diagnosis was grade 3 EC of the bilateral ovaries, involving the omentum, rectosigmoid colon, and peritoneum (FIGO stage IIIC). We also identified a 2.4 cm polypoid mass originating from the upper endocervix, with histological examination revealing several areas of EC that spread along the surface and invaded the polyp stroma. She received three cycles of post-operative adjuvant chemotherapy with paclitaxel and carboplatin. However, she presented with chest wall pain 49 months postoperatively, and chest CT and thoracic wall MRI revealed a 2.8 cm metastatic mass involving the upper sternal body and right parasternal area. She underwent complete surgical excision. Pathological examination of the mass confirmed the metastasis of ovarian EC to the sternum and rib. She is currently alive without evidence of recurrent disease, 67 months postoperatively.

*Case 2:* A 57-year-old woman was referred to our institution for the evaluation and management of uterine and adnexal masses, after visiting an outside hospital with lower abdominal pain and undergoing abdominopelvic CT and MRI. She had a history of hypertension, diabetes mellitus, pulmonary tuberculosis, and hypothyroidism. Imaging revealed a 3.4 cm endometrial mass with soft tissue density (Figure 1B) and an 8.6 cm solid and cystic left ovarian mass (Figure 1C). The retroperitoneal lymph nodes were enlarged, but pelvic and iliac chain lymph nodes were unremarkable. Peritoneal seeding or hematogenous metastasis were not observed. Radiological differential diagnoses included concurrent endometrial and ovarian carcinomas or endometrial carcinoma with ovarian metastasis. The endometrial curettage was diagnosed as grade 1 EC. Total hysterectomy with bilateral salpingo-oophorectomy, pelvic lymph node dissection, pelvic peritonectomy, appendectomy, and a small bowel resection were performed. Several enlarged mesenteric lymph nodes were detected intraoperatively but could not be completely resected. The final pathological diagnosis was synchronous endometrial grade 1 EC involving more than half of the myometrium (FIGO stage IB) and ovarian grade 3 EC involving the abdominopelvic peritoneum and small bowel (FIGO stage IIIC). We also identified a 2 cm ECP originating from the upper endocervix and showing a single microscopic focus of EC, involving the surface and superficial stroma of the polyp. Follow-up CT after three cycles of post-operative adjuvant chemotherapy with paclitaxel and carboplatin revealed a newly developed 5 cm necrotic mass invading the small bowel. She was switched from chemotherapy to pembrolizumab following metastatic recurrence and is currently alive with disease, three months postoperatively.

*Case 3:* A 57-year-old woman was referred to our institution for the evaluation and management of an incidentally detected endocervical mass. She underwent endocervical curettage at an outside hospital and was diagnosed with grade 1 EC involving ECP. Abdominopelvic CT revealed a 1 cm endometrial mass that appeared to be invading the superficial myometrium (Figure 1D). Cervical stenosis and hematometra were noted. However, no evidence of peritoneal seeding, lymph node enlargement, or distant metastasis was noted. A total hysterectomy with bilateral salpingo-oophorectomy and pelvic lymph node dissection was performed, and the specimen revealed several foci of grade 1 EC, measuring up to 0.8 cm (Figure 1E). The tumors were confined within the endometrium (FIGO stage IA). She received no further adjuvant treatment and is currently alive without evidence of recurrent disease, 24 months postoperatively.

*Case 4:* A 52-year-old woman without previous gynecological history received an endocervical polypectomy at an outside hospital and was referred to our institution for further evaluation and management. The polypectomy specimen was determined to be a grade 1 EC involving ECP. Abdominopelvic MRI revealed no visible neoplastic lesions in the cervix, endometrium, lymph node, and abdominopelvic peritoneum (Figure 1F). Under the clinical impression of MRI-invisible endometrial cancer, total hysterectomy with bilateral salpingo-oophorectomy was performed. The final pathological diagnosis was grade 1 EC limited to the endometrium (FIGO stage IA). She received no further adjuvant treatment and is currently alive without evidence of recurrent disease 15 months postoperatively.

Table 2 summarizes the clinicopathological characteristics. No patient received diagnostic or therapeutic procedure before the initial pathological diagnosis. Two ovarian ECs were advanced-stage tumors, while the two endometrial ECs were early stage diseases. The two patients who underwent debulking surgery for ovarian EC later developed metastatic recurrences despite post-operative chemotherapy, whereas the two patients who underwent total hysterectomies with bilateral salpingo-oophorectomies for endometrial EC received no further treatment and experienced no disease recurrence or metastasis.

### 3.3. Pathological Characteristics of Four ECs Involving ECP

**Figure 2:**
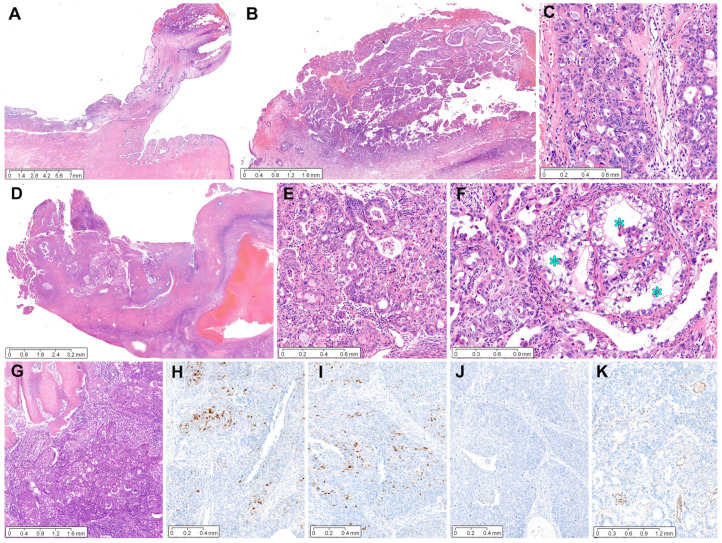
Grade 3 ovarian EC involving ECP: case 1. (**A**) A 1.7 cm pedunculated polyp arises from the endocervix. (**B**) Complex glandular proliferation involves the surface and stroma of ECP. The invasion depth measures 0.1 cm. (**C**) High-power magnification reveals poorly differentiated carcinoma displaying solid and cribriform architecture and high-grade cytological atypia. (**D**) The ovarian mass involves the surface and destructively invades the stroma. (**E**) The degree of cytological atypia and architecture are similar to those of poorly differentiated carcinoma involving ECP. (**F**) A few microscopic areas resembling clear cell carcinoma (blue asterisks) are noted in the ovarian tumor. (**G**) The sternal metastatic tumor invades the bony trabeculae and exhibits poorly differentiated carcinoma, and its histological features are similar to those of EC involving ECP (image (**C**)) and ovary (image (**E**)). (**H**–**J**) Immunohistochemical staining reveals focal and strong positivity for (**H**) estrogen receptor and (**I**) progesterone receptor and (**J**) complete absence of p53 protein expression. (**K**) Negative Wilms tumor 1 immunoreactivity excludes the possibility of serous carcinoma. (**A**–**G**), Hematoxylin and eosin staining; (**H**–**K**), immunohistochemical staining with polymer method. Original magnification: (**A**), 4×; (**B**), 40×; (**C**), 100×; (**D**), 20×; (**E**), 100×; (**F**), 200×; (**G**), 40×; (**H**), 60×; (**I**), 60×; (**J**), 60×; (**K**), 80×.

*Case 1:* A 17 mm sized ECP (Figure 2A), with a long fibrovascular stalk and elongated endocervical glands, was involved by pleomorphic EC cells. The largest dimension of EC involving ECP was 6 mm, with an invasion depth of 1 mm into the polyp stroma (Figure 2B). Poorly differentiated carcinoma involving the surface and stroma of ECP displayed solid and cribriform architecture, compatible with grade 3 EC (Figure 2C). Low-power magnification of the ovarian tumor revealed a definite involvement of the ovarian surface (Figure 2D). The primary ovarian EC showed high-grade architectural and cytological atypia, which was compatible with a grade 3 EC diagnosis (Figure 2E). A few microscopic areas resembling clear cell carcinoma were also noted (Figure 2F). The sternal metastatic tumor invaded the bony trabeculae and exhibited a poorly differentiated carcinoma, of which the histological features were similar to those of ovarian EC. Immunohistochemically, patchy p16 positivity excluded the possibility of HPV-associated EAC. The expressions of ER (Figure 2H) and PR (Figure 2I) were focal but strong in the tumor cell nuclei. Solid and cribriform architecture, high-grade nuclear atypia, and a complete lack of p53 protein expression (Figure 2J) were compatible with grade 3 EC. Negative WT1 immunoreactivity (Figure 2K) excluded the possibility of high-grade serous carcinoma.

**Figure 3:**
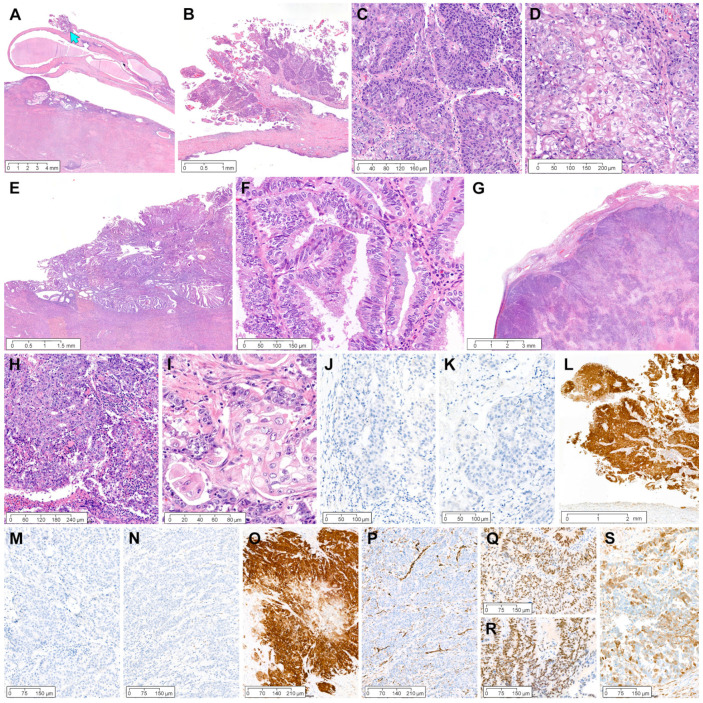
Grade 3 ovarian EC involving ECP: case 2. (**A**) The hysterectomy specimen shows an elongated ECP. EC (blue arrow) appears as an exophytic mass protruding from the surface of ECP. (**B**–**D**) EC involving ECP (**B**) superficially invades the stroma of the polyp up to 0.3 mm, (**C**) consists predominantly of solid sheets of tumor cells, and (**D**) exhibits severe nuclear pleomorphism, coarse chromatin, and prominent nucleoli. Foci of squamous differentiation are readily identifiable. (**E**) In the endometrial EC, well-differentiated glands are confluent and crowded. (**F**) Areas of mucinous differentiation are noted. (**G**) The ovarian EC demonstrates a diffuse infiltrative growth pattern with foci of geographic tumor necrosis. (**H**) The solid architecture occupies more than half of the tumor, compatible with grade 3 EC. (**I**) High-grade nuclear atypia and the presence of squamous differentiation are the same as those of EC involving ECP. (**J**–**S**) Immunohistochemically, EC involving ECP is negative for (**J**) estrogen receptor (ER) and (**K**) progesterone receptor (PR) and (**L**) positive for p16 with strong staining intensity. Ovarian EC is also negative for (**M**) ER and (**N**) PR and (**O**) uniformly and intensely positive for p16. (**P**) WT1 negativity excludes the possibility of serous carcinoma. (**Q**–**S**) Endometrial EC shows strongly positive expression for (**Q**) ER and (**R**) PR and (**S**) patchy p16 positivity. (**A**–**I**), Hematoxylin and eosin staining. (**J**–**S**), immunohistochemical staining using polymer method. Original magnification: (**A**), 10×; (**B**), 20×; (**C**), 150×; (**D**), 200×; (**E**), 25×; (**F**), 200×; (**G**), 25×; (**H**), 100×; (**I**), 400×; (**J**), 150×; (**K**), 150×; (**L**), 30×; (**M**), 100×; (**N**), 100×; (**O**), 100×; (**P**), 100×; (**Q**), 100×; (**R**), 100×; (**S**), 100×.

*Case 2:* The hysterectomy specimen incidentally showed an ECP (Figure 3A). A 19 mm elongated polyp arising in the endocervix had a 3-mm exophytic tumor protruding from the surface (Figure 3B). The tumor invaded the polyp stroma superficially, at a depth of 0.3 mm. Histologically, the EC involving ECP consisted predominantly of solid cellular sheets showing severe nuclear pleomorphism (grade 3 EC; Figure 3C), intermingled with mature squamous morules (squamous differentiation; Figure 3D). In contrast, the endometrial tumor was EC of grade 1 with mucinous differentiation (Figure 3E,F), measuring 20 mm at its largest dimension and 14 mm at its deepest invasion depth. Additionally, the patient had an ovarian mass, which was histologically compatible with grade 3 EC (Figure 3G). The presence of predominantly solid architecture (Figure 3H) and scattered areas of squamous differentiation (Figure 3I) was similar to the EC involving ECP. Moreover, the high-grade ovarian tumor, which extensively involved the pelvic and extrapelvic peritoneum, exhibited high-grade nuclear atypia, geographic necrosis, and substantial LVI. Immunohistochemical staining revealed that the EC involving ECP was negative for ER (Figure 3J) and PR (Figure 3K) but diffusely and strongly positive for p16 (Figure 3L). The immunophenotypes of the ovarian EC were identical to those of the EC involving ECP: a complete absence of ER (Figure 3M) and PR (Figure 3N) expression and uniform p16 positivity with strong staining intensity (Figure 3O). WT1 negativity in the ovarian tumor excluded high-grade serous carcinoma (Figure 3P). In contrast, endometrial EC demonstrated diffuse and strong nuclear expression for ER (Figure 3Q) and PR (Figure 3R). Patchy p16 positivity in the endometrial EC (Figure 3S) was different from intense and uniform p16 expression in the ovary and ECP. Based on the similar morphology and immunohistochemical staining results, the EC involving ECP was considered a metastatic lesion of the ovarian EC.

**Figure 4:**
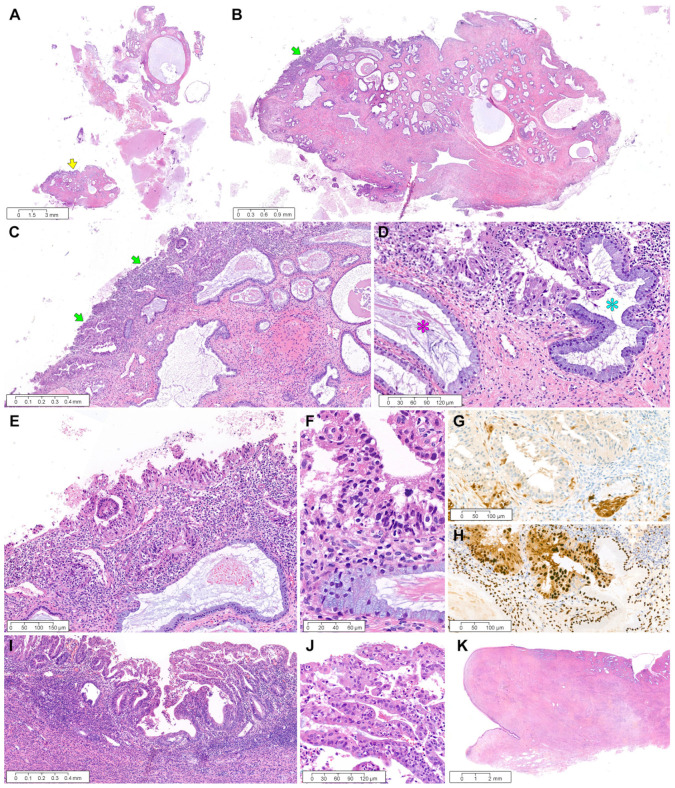
Grade 1 endometrial EC involving ECP: case 3. (**A**) The endocervical curettage specimen consists of fragmented ECP tissues, fibrin, and small endocervical tissues. One of the ECP fragments (yellow arrow) shows the tumor tissue. (**B**) The greatest dimension of EC involving the surface of ECP measures 4 mm (green arrow). The presence of thick-walled blood vessels, fibrotic stroma, and an increased number of benign endocervical glands is characteristic of ECP. (**C**) The depth of invasion into the polyp stroma (green arrows) measures 0.3 mm. (**D**) The EC cells partially involve one endocervical gland (blue asterisk), in contrast to the other uninvolved one (purple asterisk). (**E**) The tumor is associated with a stromal inflammatory reaction. (**F**) On high-power magnification, compared with non-atypical endocervical gland (lower half), the EC cells (upper half) reveal nuclear enlargement, hyperchromasia, and loss of nuclear polarity. (**G**) Patchy p16 positivity rules out the possibility of human papillomavirus-associated endocervical adenocarcinoma. (**H**) Uniform nuclear estrogen receptor immunoreactivity with intense staining intensity supports the diagnosis of grade 1 EC. (**I**) Primary endometrial EC does not invade the myometrium. (**J**) The degree of nuclear atypia is the same as that of EC involving ECP. (**K**) The cervix shows no pathological abnormality. (**A**–**F**) and (**I**–**K**), Hematoxylin and eosin staining. (**G**–**H**), immunohistochemical staining using polymer method. Original magnification: (**A**), 2.5×; (**B**), 10×; (**C**), 40×; (**D**), 150×; (**E**), 100×; (**F**), 400×; (**G**), 100×; (**H**), 100×; (**I**), 40×; (**J**), 150×; (**K**), 4×.

*Case 3:* Endocervical curettage revealed a few pieces of endocervical tissue showing cystically dilated glands, fibrous stroma, and endocervical tissue fragments (Figure 4A). An 11 mm ECP was involved with the superficially invading tumor at the polyp surface (Figure 4B). This tumor measured 4 mm at its greatest dimension and 0.3 mm at its deepest invasion depth (Figure 4C). Foci of endocervical glandular extension were occasionally noted (Figure 4D). A well-differentiated glandular proliferation was associated with stromal inflammatory reaction (Figure 4E), and the tumor cells exhibited nuclear hyperchromasia and mild pleomorphism (Figure 4F). Immunohistochemically, patchy p16 positivity eliminated the possibility of HPV-associated EAC (Figure 4G). Uniform estrogen receptor immunoreactivity with intense staining intensity supported the diagnosis of grade 1 EC (Figure 4H). The endometrial tumor was an 8 mm EC without myometrial invasion (Figure 4I). The degree of cytological and architectural atypia matched that of the EC involving ECP (Figure 4J). No remarkable lesion was identified in the cervix (Figure 3K).

**Figure 5:**
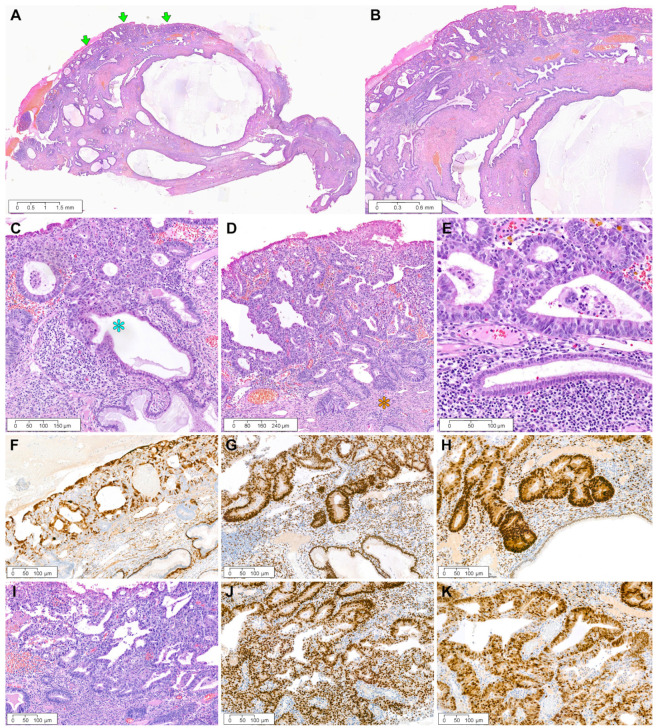
Grade 1 endometrial EC involving ECP: case 4. (**A**) The polypectomy specimen demonstrates the tumor tissue (green arrows) involving the surface. (**B**) Complex and crowded glandular architecture spreads along the surface and superficial stroma of ECP. (**C**) The EC cells partially involve one endocervical gland (blue asterisk). (**D**) Back-to-back arrangement (orange asterisk) and a lack of the intervening stroma are compatible with grade 1 EC. (**E**) High-power magnification reveals that the tumor glands (upper half) display nuclear stratification, enlargement, hyperchromasia, and loss of nuclear polarity. There is also a non-atypical endocervical gland (lower half). (**F**–**H**) Immunohistochemical staining reveals (**F**) patchy p16 positivity and diffuse and strong expression for (**G**) estrogen and (**H**) progesterone receptors. (**I**–**K**) Primary endometrial EC exhibits identical (**I**) histological features and immunoreactivities for (**J**) estrogen and (**K**) progesterone receptors to EC involving ECP. (**A**–**E**) and (**I**), Hematoxylin and eosin staining; (**F**–**H**), (**J**), and (**K**), immunohistochemical staining using polymer method. Original magnification: (**A**), 15×; (**B**), 20×; (**C**), 60×; (**D**), 40×; (**E**), 200×; (**F**), 100×; (**G**), 100×; (**H**), 100×; (**I**), 100×; (**J**), 100×; (**K**), 100×.

*Case 4:* The polypectomy specimen revealed a 16 mm ovoid polyp with a short, slender stalk comprising a centrally dilated glandular lumen, peripherally stretched glands, and fibrotic stroma (Figure 5A). Complex and crowded glands were spread along the polyp surface (Figure 5B), and the superficial stroma of ECP was invaded by the tumor at a depth of 1 mm and a dimension of 10 mm. Endocervical glandular extension (Figure 5C) and stromal inflammatory infiltrates (Figure 5D) were present. The tumor cells displayed mild-to-moderate nuclear pleomorphism and enlargement (Figure 5E). Immunohistochemical staining revealed patchy p16 positivity (Figure 5F) and diffuse and strong expression for ER (Figure 5G) and PR (Figure 5H). The histological features were compatible with a grade 1 EC diagnosis. We found a 5-mm endometrial tumor without myometrial invasion or LVI. This endometrial lesion was morphologically (Figure 5I) and immunophenotypically (Figure 5J,K) identical to the EC involving ECP.

Table 3 summarizes the immunohistochemical staining results. The histological type of malignancy involving ECP was EC in all four cases, with two grade 3 ovarian ECs and three grade 1 endometrial ECs. One of the ovarian tumors exhibited foci of squamous differentiation, and one patient (case 2) had concomitant ovarian and endometrial ECs. Three cases displayed extension into the endocervical glands embedded in the polyp stroma. Although all tumors invaded the polyp stroma up to 1 mm of depth, no LVI was noted. The resection margin status was evaluated in two patients who underwent polypectomies. Both patients showed a negative resection margin, but the safety distance was < 1 mm in one case. No evidence of endometriosis, hyperplasia without atypia, or AH/EIN was observed in ECP, whereas all primary endometrial ECs were associated with AH/EIN and all ovarian tumors co-existed with endometriotic cysts. Interestingly, the three primary tumors (cases 1, 3, and 4) showed no LVI or lymph node metastasis. Detailed histopathological features of EC involving ECP are as follows.

## 4. Discussion

To the best of our knowledge, no previous cases of the synchronous occurrence of endometrial or ovarian EC involving ECP have been reported. Our study described four such cases. In cases 3 and 4, we diagnosed EC involving ECP as a metastatic lesion from endometrial EC based on the following morphological features: (1) same histological features and immunohistochemical staining results between the EC involving ECP and the endometrium; (2) no involvement of the lower uterine segment and cervical stroma; (3) no LVI; (4) no precursor lesion (benign endometriosis and/or endometrial hyperplasia without atypia) in ECP; and (5) no premalignant lesion in ECP. Most endometrial ECs with the direct involvement of the cervix present as large tumors or with an epicenter in the lower uterine segment [31]. In some cases, the tumor cells spread to the endocervix along the surface or glandular epithelium. LVI is also one of the ways by which EC may involve the cervix. However, our two endometrial EC patients had no evidence of tumor involvement or LVI in the lower uterine segment, cervical stroma, and endocervical mucosa. EC of the uterine cervix is classified as one of the HPV-independent EACs according to the updated 2020 World Health Organization Classification [16]. Primary cervical EC may arise from benign endometriosis involving ECP and progress to AH/EIN and eventually EC. The absence of benign endometriosis, endometrial hyperplasia without atypia, or AH/EIN in ECP and adjacent non-polypoid endocervical tissue did not support the possibility of HPV-independent endometrioid-type EAC. Finally, we considered implantation metastasis as a possible mechanism of the ECP involvement of endometrial EC. Iatrogenic tumor implantation is a condition that results from various medical procedures used during the diagnosis or treatment of a malignancy [32]. In one study, of the 176 patients who underwent endometrial curettage before hysterectomy, 9 (5.1%) were found to have cervical implantation metastasis [33]. Moreover, endometrial carcinomas are well known to have an implantation capacity, which is the ability of tumor cells detached from the primary endometrial tumor to migrate to the peritoneal cavity through the fallopian tubes for implantation on the peritoneal surface, resulting in peritoneal, cervical, or vaginal implantation [34]. In a study by Stewart et al. [32], tumor cell emboli within the tubal lumina were identified in 26% and 3% of high- and low-grade endometrial carcinomas, respectively. Since we did not observe EC cells migrating through the endometrial cavity and endocervical canal experimentally, we could not clearly clarify the pathogenetic mechanism by which the endometrial EC involves ECP in this study. However, based on the absence of tumor involvement or LVI in the lower uterine segment, cervical stroma, and endocervical mucosa, the possibility that the tumor cells may have been implanted on the ECP surface through intrauterine migration can be considered. Even though our two endometrial EC patients had not undergone any previous diagnostic or therapeutic procedures prior to endocervical polypectomy (case 3) or endometrial curettage (case 4), it is reasonable to assume that the tumor cells may have adhered to the erosive and inflamed surface of the continuously compressed and irritated ECP within the narrow endocervical canal.

In cases 1 and 2, the histological features of the grade 3 EC involving ECP and the ovarian tumors were the same. Endometrial or cervical metastases of ovarian cancer are rarer than ovarian metastases of endometrial or cervical cancer [31,35]. Our finding of ovarian EC metastasizing to the ECP and spreading along the polyp surface or superficially invading into the polyp stroma appears to be novel. Substantial LVI was observed in case 2, whereas there was no LVI in the ovary and ECP in case 1. The latter case presented an unusual phenomenon, as the tumor cells must have migrated in the opposite direction, from the ovary and peritoneal cavity through the endometrium to the cervix. Ovarian EC cells on the ovarian surface or around fimbria may migrate through the tube and endometrial cavity to the endocervical canal [31,36]. Two cases of ovarian EC were initially advanced stage, with neoadjuvant chemotherapy being performed in one case. As a result of the pathological examination of the debulking specimen, both cases were stage IIIC and high-grade, and one (case 1) was p53-abnormal EC. In case 1, metastases to the sternum and rib occurred 49 months after post-operative adjuvant chemotherapy, and in case 2, metastasis was observed in the mesentery after three cycles of adjuvant chemotherapy. The presence of small metastases of ovarian EC to ECP less than 1 cm did not affect the treatment decisions or outcomes of the patients with the aggressive, advanced-stage, high-grade ovarian EC. Our findings did, however, reflect the high oncogenic aggressiveness shown in the migratory and metastatic ability of these tumors.

Case 2 demonstrated concurrent endometrial and ovarian ECs and multiple metastatic lesions in the abdominopelvic peritoneum and small bowel. The following differential diagnoses were initially considered: (1) adnexal and peritoneal involvement of endometrial cancer, (2) peritoneal and endometrial involvement of ovarian cancer, and (3) synchronous endometrial and ovarian cancers with peritoneal extension from either endometrium or ovary. The histology (high-grade EC with squamous differentiation) and immunophenotype (hormone receptor negativity and diffuse, strong p16 positivity) of the peritoneal metastatic lesions were the same as those of the ovarian tumors. However, the endometrial tumors were low-grade EC with mucinous differentiation, showing uniform hormone receptor positivity and p16 patchy positivity. Based on these results, it was determined that the possibility of synchronous stage IIIC ovarian cancer (with extrapelvic peritoneal metastases) and stage IB endometrial cancers was high. The EC observed in ECP also had the same morphology and immunophenotype as the ovarian EC but were different from the endometrial EC. Additionally, endometriosis, endometrial hyperplasia without atypia, and AH/EIN, suggesting the possibility of primary HPV-independent endometrioid-type EAC, were absent in ECP. Therefore, the EC involving ECP was reasonably considered a metastasis of the ovarian EC.

Several clinical implications of the involvement of ECP by endometrial or ovarian EC were considered. First, in case 3, grade 1 EC involving ECP was diagnosed in the curettage of an incidentally detected endocervical mass. In case 4, grade 1 EC was found in an endocervical polypectomy specimen, although an endometrial lesion was not suspected clinically. As in these cases, when metastatic tumors involving ECP were first detected where the primary endometrial EC was not known, the lesions helped detect endometrial cancer at the early stages. Second, in cases 3 and 4, where EC invaded the polyp stroma to a depth of less than 1 mm, the lesions could not be considered cervical stromal extensions, a FIGO stage II finding of endometrial cancer. In other words, adjuvant radiation therapy, the standard treatment for stage II endometrial cancer, was not required. The treatment could differ, in the case of case 3 potentially being misinterpreted as an endometrioid-type EAC involving ECP, for instance. The gynecologists may have considered whether to perform radical hysterectomy with bilateral salpingo-oophorectomy and pelvic lymph node dissection, the standard treatment for cervical cancer, or simply a total hysterectomy with bilateral salpingo-oophorectomy, considering that there was no visible uterine lesion and lymph node metastasis on MRI. Third, when stratified according to the stage, grade, and myometrial invasion, no statistically significant differences in the recurrence rate between patients with or without cervical implantation metastasis exist [33], indicating that cervical implantation metastasis does not appear to alter prognoses or require specific treatment. In this study, since the ovarian ECs of cases 1 and 2 were advanced-stage diseases, the ECP tumor did not affect the treatment. In addition, the ECP involvement by endometrial EC in cases 3 and 4 did not affect the treatment guideline in these cases, as the ECP was removed with the total hysterectomies.

**Figure 6:**
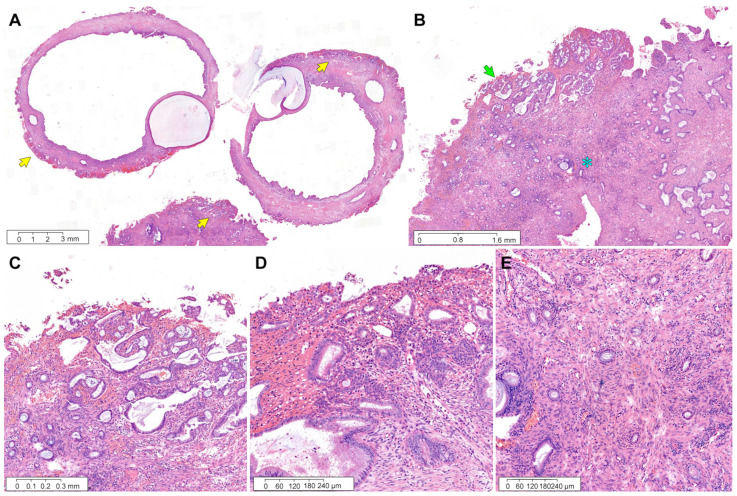
SM involving ECP. (**A**) ECP of case 1 reveals areas of SM and reactive glandular proliferation (yellow arrows). Some cystically dilated glands are present. (**B**) In addition to the immature SM (green arrow), small glands are embedded in the fibrotic stroma (blue asterisk). (**C**) The endocervical glands in SM lesions vary in size and shape. (**D**) Metaplastic squamous epithelium possesses small, bland nuclei and scant cytoplasm and grows beneath the pre-existing endocervical epithelium. There is no complex glandular proliferation, solid architecture, cribriforming, or high-grade cytological atypia. (**E**) The stroma exhibits fibrosis and mixed chronic inflammatory infiltrates. (**A**–**E**), Hematoxylin and eosin staining. Original magnification: (**A**), 15×; (**B**), 40×; (**C**), 100×; (**D**), 150×; (**E**), 120×.

Differential diagnoses of EC involving ECP include reactive endocervical lesions, including squamous metaplasia (SM) and microglandular hyperplasia (MGH), and HPV-associated usual-type EAC. ECPs are commonly accompanied by SM, MGH, and chronic inflammation. Immature SM is characterized by evenly spaced, small, round nuclei and dense, scant cytoplasm involving the superficial epithelium and glands of ECP. Case 1 revealed SM involving ECP on the non-neoplastic areas of the polyp (Figure 6A–C). SM had no mitotic activity or significant nuclear or architectural atypia (Figure 6D). The intervening stroma displayed fibrosis and chronic inflammation (Figure 6E). MGH consists of closely packed, small glands with mucin-containing epithelium in the background of mildly inflamed stroma. Despite a compact glandular proliferation, MGH exhibits no nuclear pleomorphism or architectural abnormality. HPV-associated usual-type EAC is the most common type of EAC, with apical mitoses and apoptotic bodies readily identifiable by scanning or low-power magnification. Block p16 positivity is the hallmark of HPV-associated EAC, but all our cases showed patchy p16 immunoreactivity.

## 5. Conclusions

In summary, we evaluated the histological types and prevalence of gynecological tumors co-existing with ECP. We observed that 69 of the 429 ECPs (15.9%) were found to be associated with premalignant or malignant lesions of the uterine cervix, endometrium, and ovary. Of these, four (0.9%) ECPs were involved by endometrial or ovarian ECs. We investigated the clinicopathological characteristics of the four cases of ECP that were involved by EC. In two cases of ovarian EC, EC involving ECP exhibited similar morphology and immunohistochemical staining results as those of advanced-stage ovarian EC. In two cases of endometrial EC, the histological and immunophenotypical features of the EC involving ECP were identical to those of the primary endometrial tumor, despite the lack of tumor involvement in the myometrium, lower uterine segment, and cervical stroma as well as the absence of LVI and lymph node metastasis. In all cases, no evidence of benign endometriosis, endometrial hyperplasia without atypia, or AH/EIN within ECP or the adjacent endocervical tissue was noted. Based on clinical history, histological features, and immunohistochemical staining results, we concluded that they were metastatic from the endometrial or ovarian ECs, as possible implantation metastases. The determination of the type and origin of metastatic tumors is an important and potentially challenging area in pathology, as it affects the clinical decision and patient management. The site of origin is best determined by correlating clinical and pathologic findings. The occurrence of endometrial and ovarian carcinomas metastatic to ECPs is a rare phenomenon. To the best of our knowledge, we described the first synchronous occurrence of EC involving ECP and endometrial or ovarian EC. Awareness of these unusual phenomena is vital in proper diagnosis and clinical practices.

## Figures and Tables

**Table 1:** The histological types and prevalence of gynecological tumors co-existing with ECP.

Origin	Relationship with ECP	Histological Type	Number of Cases (Prevalence)
Uterine cervix	Involving ECP	HSIL	11 (2.6%)
		SCC	3 (0.7%)
		AIS	2 (0.5%)
		SMILE and HSIL	1 (0.2%)
	Separate	SCC	2 (0.5%)
		AIS and HSIL	1 (0.2%)
EM	Involving ECP	EC	2 (0.5%)
	Separate	EC	14 (3.3%)
		AH/EIN	6 (1.4%)
		EC and CCC	1 (0.2%)
		SC	1 (0.2%)
Ovary	Involving ECP	EC	2 (0.5%)
	Separate	HGSC	14 (3.3%)
		CCC	3 (0.7%)
		EC	2 (0.5%)
		MC	2 (0.5%)
		LGSC	1 (0.2%)
Total			68 (15.9%)

AIS: Adenocarcinoma in situ; AH/EIN: atypical hyperplasia/endometrial intraepithelial neoplasm; CCC: clear cell carcinoma; EC: endometrial carcinoma; EM: endometrium; HGSC: high-grade serous carcinoma; HSIL: high-grade squamous intraepithelial lesion; LGSC: low-grade serous carcinoma; MC: mucinous carcinoma; SCC: squamous cell carcinoma; SMILE: stratified mucin-producing intraepithelial lesion.

**Table 2:** Clinicopathological characteristics of four patients with EC involving ECP.

Case No	1	2	3	4
Age	33 years	57 years	57 years	52 years
Imaging finding	8.6-cm solid and cystic bilateral ovarian masses; borderline-sized pelvic and retroperitoneal lymph nodes; peritoneal carcinomatosis	8.6-cm solid and cystic left ovarian mass; 3.4-cm EM mass; enlarged retroperitoneal lymph nodes; peritoneal carcinomatosis	1-cm EM mass; no lymph node enlargement; no peritoneal seeding	No identifiable EM lesion; no lymph node enlargement; no peritoneal seeding
Clinical impression	Ovarian cancer	Concurrent ovarian and EM cancers	EM cancer	MRI-invisible EM cancer
Neoadjuvant chemotherapy	Paclitaxel-carboplatin (three cycles)	Not received	Not received	Not received
Surgical procedure	TH, BSO, PLND, PALND, low anterior resection, omentectomy	TH, BSO, PLND, small bowel resection, appendectomy, omentectomy, peritonectomy	TH, BSO, PLND	TH, BSO
Final pathological diagnosis	Stage IIIC grade 3 EC (ovary)	Stage IIIC grade 3 EC (ovary); stage IB grade 1 EC (EM)	Stage IA grade 1 EC (EM)	Stage IA grade 1 EC (EM)
Greatest dimension of ECP	17 mm	19 mm	11 mm	16 mm
Greatest dimension of EC	6 mm	3 mm	4 mm	10 mm
Invasion depth into polyp stroma	1 mm	0.3 mm	0.3 mm	1 mm
Polypectomy resection margin involvement (safety distance)	NA	NA	Absent (5 mm)	Absent (<1 mm)
Post-operative treatment	Paclitaxel-carboplatin (three cycles)	Paclitaxel-carboplatin (three cycles)	Not received	Not received
Post-operative recurrence	Bone (sternum and rib)	Mesentery	Absent	Absent
Disease-free survival	49 months	3 months	24 months	15 months
Treatment for recurrence	Complete surgical excision	Pembrolizumab (regimen change)	Not received	Not received
Survival status	Alive	Alive	Alive	Alive
Overall survival	67 months	3 months	24 months	15 months

BSO: Bilateral salpingo-oophorectomy; EM: endometrium; MRI: magnetic resonance imaging; PALND: para-aortic lymph node dissection; PLND: pelvic lymph node dissection; TH: total hysterectomy.

**Table 3:** The histological types and prevalence of gynecological tumors coexisting with ECP.

Case No	1		2			3		4	
	ECP	Ovary	ECP	Ovary	EM	ECP	EM	ECP	EM
ER	FSP	FSP	Neg	Neg	DSP	DSP	DSP	DSP	DSP
PR	FSP	FSP	Neg	Neg	DSP	FSP	FSP	DSP	DSP
p16	PP	PP	DSP	DSP	PP	PP	PP	PP	PP
p53	Mutant (CA)	Mutant (CA)	WT	WT	WT	WT	WT	WT	WT
Wilms tumor 1	Neg	Neg	Neg	Neg	Neg	NA	NA	NA	NA

CA: Complete absence; DSP: diffuse strong positive; ER: estrogen receptor; FSP: focal strong positive; Neg: negative; PP: patchy positive; PR: progesterone receptor; WT: wild type.

## Data Availability

Not applicable.
